# Mechanisms of mitochondrial dysfunction in ovarian aging and potential interventions

**DOI:** 10.3389/fendo.2024.1361289

**Published:** 2024-04-17

**Authors:** Wenhan Ju, Yuewen Zhao, Yi Yu, Shuai Zhao, Shan Xiang, Fang Lian

**Affiliations:** ^1^ The First Clinical Medical College, Shandong University of Traditional Chinese Medicine, Jinan, China; ^2^ CReATe Fertility Centre, Toronto, ON, Canada; ^3^ Department of Reproduction and Genetics, Affiliated Hospital of Shandong University of Traditional Chinese Medicine, Jinan, China

**Keywords:** oocyte, aging, mitochondria, fertility preservation, mechanism

## Abstract

Mitochondria plays an essential role in regulating cellular metabolic homeostasis, proliferation/differentiation, and cell death. Mitochondrial dysfunction is implicated in many age-related pathologies. Evidence supports that the dysfunction of mitochondria and the decline of mitochondrial DNA copy number negatively affect ovarian aging. However, the mechanism of ovarian aging is still unclear. Treatment methods, including antioxidant applications, mitochondrial transplantation, emerging biomaterials, and advanced technologies, are being used to improve mitochondrial function and restore oocyte quality. This article reviews key evidence and research updates on mitochondrial damage in the pathogenesis of ovarian aging, emphasizing that mitochondrial damage may accelerate and lead to cellular senescence and ovarian aging, as well as exploring potential methods for using mitochondrial mechanisms to slow down aging and improve oocyte quality.

## 1 Introduction

**Figure 1:**
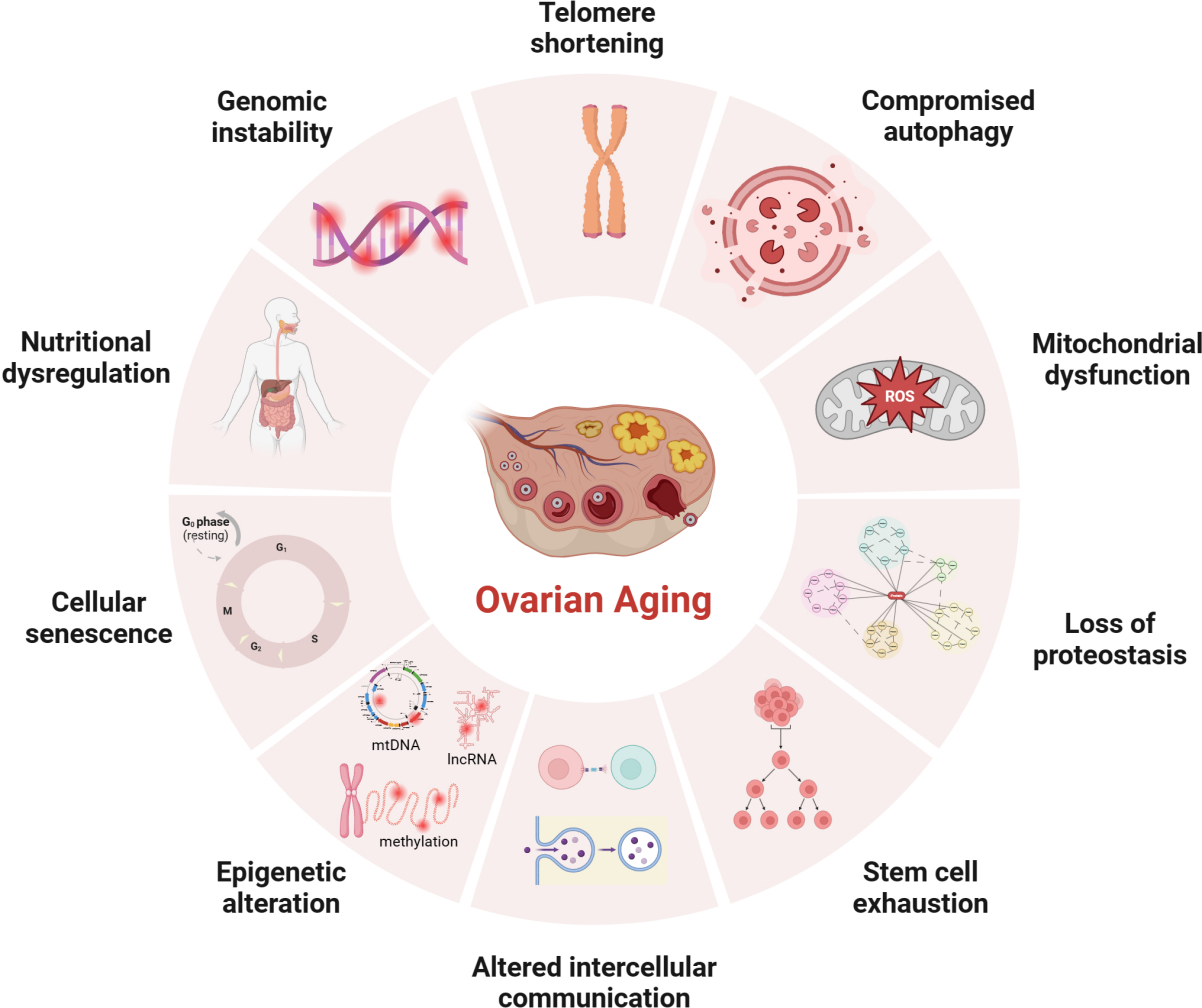
Potential mechanisms of ovarian aging (the figure was created with BioRender.com) Telomere shortening: Once telomere length becomes critically short, cellular senescence and apoptosis may occur during ovarian aging in mammals (7, 8); Compromised autophagy: The decrease of autophagic activity with age, likely leads to the accumulation of damaged macromolecules and organelles (9, 10); Mitochondial dysfunction: Mitochondial factors are explained further under subheadings in the text; Loss of proteostasis: Loss of proteostasis may damage the stability of microtubules and the integrity of meiosis in naturally aged mice oocyte (11); Stem cell exhaustion: Due to germ-line stem cells do not exist post-natally in female mammals (12–14), the reproductive potential of the ovaries will continue to decline after birth; Altered intercellular communication: There is an Increase In preantral follicles atresia in mice with suppression of intercellular junctions, which may be the cause of premature ovarian failure (15, 16); Epigenetic alteration: Studies have revealed the occurrence of epigenetic changes in the cells within the ovaries as age increases, including abnormal DNA methylation (17), histone modifications (18), and non-coding RNA-regulated modifications (19, 20); Cellular senescence: With the dysfunction of senescent cell, limited mitochondrial energy production, increased apoptosis of ovarian granulosa cells (21), increased inflammatory cytokines secretion (22) etc., would alter the microenvironment of follicle growth and affecting the quality and quantity of oocyte; Nutritional dysregulation: Dietary factors (23), low energy availability and high fat diet can increase the risk of POI; Genomic instability: In aging oocyte deceased efficacy of DNA repair mechanisms could cause low potentiation of anti-oxidant buffering and promote cell death (24).

The average age of primiparous women has been gradually increasing since the 21st century, which is known to be negatively associated with reproductive outcomes (1–3). The advancement of assisted reproductive technology can compensate for the age-related decline in fertility, but evidence suggests that stopping or reversing the biological aging process is impossible (4, 5). Patients with certain genetic and autoimmune diseases, or women with excessive dieting, long-term radiation interference, or postoperative chemotherapy for cancer, may experience a premature decline in ovarian function (6), resulting in early-onset ovarian dysfunction, also called premature ovarian insufficiency (POI). Age-related infertility is mainly related to the decreased quantity and quality of oocyte that limit a woman’s ability to conceive. Studies using animal models revealed several cellular and genetic dysfunctions are causally related or correlated with aging. Although the mechanism of ovarian aging remains unclear, possible explanations for female ovarian aging are summarized in **Figure 1**.

Mitochondrial dysfunction provides a clue for explaining ovarian aging. Mitochondria is a kind of organelle with a double membrane structure and a diameter of 0.5~1.0 μm, containing limited Genetic material. Mitochondria is controlled by the mitochondrial and nuclear genomes. Human mitochondrial DNA (mtDNA) is a circular DNA molecule containing 16569 base pairs. All 13 proteins encoded by mtDNA are structural components of the electron transport chain (ETC), which assemble with nuclear-encoded proteins and become crucial components of the oxidative phosphorylation(OXPHOS) process. Mitochondria is the sites for oxidative metabolism in eukaryotes, and is the sites where sugars, fats, and amino acids ultimately oxidize and release energy. The common pathway responsible for the final oxidation of mitochondria is the tricarboxylic acid (TCA) cycle and OXPHOS, which correspond to the second and third stages of aerobic respiration, respectively. The glycolysis completed in the cytoplasmic matrix and the TCA cycle completed in the mitochondrial matrix produce high-energy molecules such as reduced nicotinamide adenine dinucleotide (NADH) and reduced flavin adenine dinucleotide (FADH2). The role of OXPHOS in this step is to use these substances to reduce oxygen and release energy to synthesize adenosine triphosphate (ATP).

A big difference from somatic cells is that the number of mitochondria and the copy number of mtDNA in human mature oocyte are very high (6). These mitochondria are produced during the oogenesis stage, and the mitochondrial replication is believed to remain quiescent until the embryo reaches the blastocyst stage (25, 26). Using transcriptome sequencing, differential expression of mitochondrial-related genes was found by comparing the oocyte of young women and women with advanced age (27–30). It is reported that the increase in maternal age significantly decreased the number and quality of mitochondria in aging oocyte (31, 32). After ovarian aging, the morphology of mitochondria changed significantly, showing an increase in the number of swollen and vacuolated mitochondria (33–37). But ovarian functions can be partially restored by supplementing healthy mitochondria or improving mitochondrial function (38, 39), such as reducing the risk of mitochondrial oxidative damage caused by free radicals, reducing the expression of mitochondrial apoptosis-related proteins (40), and stimulating mitochondrial autophagy enhancers (41). The current manuscript reviews and summarizes the mechanism of mitochondrial damage in ovarian aging to explore possible intervention measures for improving and protecting the fertility of women with advanced age of childbearing age.

## 2 Mitochondrial dysfunction leads to ovarian aging

### 2.1 The key role of mitochondrial oxidative stress damage in ovarian aging

The disruption of oxidative and antioxidant balance leads to excessive oxidative stress(OS), resulting in irreversible damage of ovarian. Reactive oxygen species (ROS) are byproducts resulting from energy production in the mitochondrial electron transfer chain to generate ATP. When the human body ages or is subjected to various harmful stimuli, excessive production of ROS and reactive nitrogen species (RNS) can disrupt the antioxidant defense barrier, trigger cellular OS response. The emergence of mitochondrial OS may be a mechanism leading to infertility, which is not unfamiliar to us. The high OS level detected in the human ovary is associated with follicular atresia, low fertilization potential of oocyte, the risk of aneuploidy, and sub-fertility (42, 43). Numerous studies have shown that during ovarian aging, the antioxidant enzymes in granulosa cells(GCs), and follicular fluid, such as superoxide dismutase (SOD), catalase (CAT), and Glutathione peroxidase (GSH-Px), were significantly reduced (44, 45). OS reaction is regarded as a specific initiator for oocyte aging (46), directly attacking the mitochondrial membrane and destroying the mitochondrial OXPHOS, resulting in reduced ATP production. Excessive ROS production directly affecting the target of the signaling pathway and acting as a second messenger interacting with intermediate reaction steps (47). ROS reduces the expression of isocitrate dehydrogenase 1 (IDH1) by activating the mitogen-activated protein kinase (MAPK) signaling pathway (48), activates the p53-SIAH1-TRF2 axis to induce telomere shortening (49), and promotes the aging of GCs. In addition to aging, GCs apoptosis is related to follicular atresia, which is an important mechanism of ovarian aging. Research has found that the strong oxidant H_2_O_2_ passes through HIF-1α signaling pathway to stimulates apoptosis of GCs (50). Peroxiredoxin 2(PRX2)deficiency accelerated age-related ovarian failure through the ROS-mediated JNK pathway in mice, and increased numbers of apoptotic cells (51). Another study showed that Ginsenoside Rb1 inhibits age-related GCs oxidative damage by activating Akt phosphorylation at Ser473 and by further interaction with FOXO1 (44). Therefore, the increase of OS plays an important role in the development of ovarian aging. The use of antioxidants to minimize OS may affect improving ovarian function women with advanced age (41, 43).

It is worth noting that when OS occurs, mitochondria will respond. Nuclear respiratory factors (NRF) can sense OS in the mitochondrial matrix and be activated. NRF1/2 can regulate the formation of mitochondrial respiratory chain complexes, transcription and replication of mtDNA. Loss of full-length NRF1 led to a dramatic increase in ROS and oxidative damages (52). In addition, SIRT1/3 also acts as a sensor by activating PGC1α- NRF1 promotes mitochondrial synthesis and coordinates cellular defense against ROS invasion (53, 54). Unfortunately, current research has found that NRF1, SIRT1, and SIRT3 are all downregulated in the ovaries of reproductive aging (55–57), indicating a decrease in the ability of mtDNA transcription and replication (mitochondrial synthesis). The antagonist response orchestrated by SIRT1 in oocyte seems to decrease with aging (53). When ROS accumulation exceeds the critical point, OS will further induce cell aging or apoptosis, affecting oocyte quality.

In summary, the increase of OS plays an important role in the development of ovarian aging by disrupting OXPHOS, inducing telomere shortening, and stimulating cell apoptosis (46–48). At the same time, the expression of molecules that receive ROS signals to activate mitochondrial synthesis is downregulated in the aging ovaries (55–57). The insufficient transcription and replication ability(mitochondrial synthesis) of mtDNA leads to a decreased ability to resist ROS in the cells.

### 2.2 Changes in mitochondrial genome during ovarian aging

#### 2.2.1 The decrease in mtDNA quantity leads to a decrease in the quality of aging oocyte

When OS occurs within cells, mtDNA transcription and replication are driven to synthesize new mitochondria. Unfortunately, it has been found that mtDNA copy number decreases with ovarian aging (58, 59). The mtDNA is compacted into dense mitochondrial nucleoids. The mitochondrial transcription factor A (TFAM) binds to mtDNA and regulates mtDNA transcription activation *in vivo* by recruiting mitochondrial RNA polymerase (POLRMT) and mitochondrial transcription factor B (TFBM) for mtDNA replication (58). Current research has found that female individuals with TFAM recessive missense mutation showed the clinical phenotype of POI (59). Evidence up to date indicated the decreasing pattern of mitochondrial quantity and mtDNA content in mammalian oocyte with aging (31, 60, 61). In addition, a significant decrease in the copy number of mtDNA was found in unfertilized and degenerated oocyte of women with advanced age, with a notable positive correlation existing between the cytoplasmic volume of blastomeres from embryos and the mtDNA copy number (35). The decrease in mtDNA copy number is consistent with the difficulty in conceiving in patients with reproductive aging. The mtDNA copy number is an indicator of oocyte maturation and fertilization potential (62). The optimal mtDNA copy number and sufficient ATP level (at least 2pMol) are prerequisites for normal follicular development and maturation to ensure the good developmental potential of the fertilized blastocyst (63, 64).

On the other hand, the quality of oocyte is regulated by GCs (65). GCs include two types: mural granulosa cells(MGCs) and cumulus cells (CCs). Current research has found that, women with moderate expression of TFAM in the cytoplasm of human follicular fluid GCs exhibit better results in IVF (66). The mtDNA content of CCs and oocyte in women with diminished ovarian reserve (DOR) significantly decreased, while the quality of oocyte was positively correlated with the expression of TFAM mRNA in CCs (60). The copy number of mtDNA in GCs in POI patients and low prognosis in patients classified by POSEIDON negatively correlate with age (67). Due to the crucial importance of maintaining stable mtDNA copy number for maintaining mitochondrial function and cell growth (68), the decrease in mtDNA copy number in GCs is closely related to the occurrence of excessive cell apoptosis (69). Excessive apoptosis of GCs can lead to follicular atresia and decreased oocyte quality.

In summary, a significant decrease in the copy number of mtDNA in oocyte and GCs was found in individuals with reproductive aging (35, 60, 67). The decrease in mtDNA copy number is associated with a decrease in oocyte fertilization potential. In this context, some researchers have carried out mitochondrial transplantation techniques with the aim of increasing the number of healthy mitochondria in the cells, resulting in improved oocyte quality and pregnancy rates (70, 71). However, due to the small sample size of the study, further expansion is still needed to confirm the findings. Due to the high number of mitochondria and copy number of mtDNA in mature human oocyte (6). The decrease in mtDNA copy number may only affect one aspect of oocyte quality, and mtDNA mutations cannot be ignored.

#### 2.2.2 The significant increase in mtDNA instability leads to a decrease in the quality of aging oocyte

The “mitochondrial aging theory” hypothesizes that mtDNA undergoes sustained oxidative damage, leading to the accumulation of harmful mutations (72). ROS formation takes place in close proximity to mtDNA, which lacks the protective measures of histone complexes and efficient DNA repair mechanisms, in contrast to nuclear DNA. Consequently, this vulnerability results in a heightened mutation rate (73). In situations such as aging, obesity, or chronic inflammatory stimulation, ROS production escalates, which renders mtDNA more susceptible to mutations (74, 75). The instability of mtDNA is manifested in various means, including mutations, base oxidation modifications, single-strand breaks, and so on. It has been found that harmful mtDNA mutations gradually accumulate in aging human tissues (76, 77). In addition, multiple studies have demonstrated that mutations in mtDNA encoding genes may lead to mitochondrial dysfunction and play a positive role in the pathogenesis of POI (28–30).

However, a few studies found no correlation between mtDNA deletion, rearrangement, and mutations of human oocyte and maternal age (75, 78–80). In their studies, the mtDNA mutation rate of oocyte was as high as 28~50.5%, and that of CCs was as high as 66% (78–80). The heteroplasmy created by mtDNA mutations are common in oocyte. The mtDNA bottleneck genetic mechanism during generational transmission is predicted to filter out the mitochondria population with mtDNA mutations effectively (81, 82). The difference in representative phenotypes and lower recurrence risk among women carrying heterogeneous mtDNA mutations demonstrate the effectiveness of the bottleneck theory (83, 84). Additionally, the number of primordial follicles in female mammals is fixed at birth, and there is no regeneration or renewal of the follicular pool after birth. It is unclear whether the prolonged quiescent stage before the resumption of meiosis could be affected by a hypoxic environment to undergo mtDNA mutations. In addition, during the oocyte’s maturation, there is a surge in mtDNA quantity and redox reactions (6), due to excessive ROS can also lead to mtDNA mutations, making it difficult to evaluate the occurrence time of mtDNA mutations.

Besides, nuclear encoded mitochondrial Helicase Twinkle (TWNK) and mitochondrial DNA polymerase γ (POLG) are essential proteins for mtDNA proofreading and fidelity (85–87). In humans, individuals with TWNK mutations exhibit Perrault syndrome (ovarian hypoplasia) (88), individuals with POLG mutations exhibit premature aging (89), genome wide association studies(GWAS) has found that POLG is associated with female menopause (89), and individuals with DOR have decreased POLG mRNA expression in CCs (90). POLG mutant mice showed a decrease in the number of mitochondria, abnormal mitochondrial distribution (aggregation and clustering), lower NADH/NAD+ redox ratio and weaker energy production, and a corresponding decrease in fertility (91, 92). The above research highlights the insight that mutations in nuclear coding genes lead to ovarian aging by affecting the stability of mtDNA.

In summary, many studies on mitochondrial gene expression have shown a link between decreased mtDNA copy number, accumulated mtDNA mutations, and ovarian failure. Genes are internal factors that determine the normal operation of mitochondria. The instability of the mtDNA genome will lead to dysfunction of executing proteins within mitochondria, which in turn will lead to a decrease in mitochondrial function. In fact, there is an endogenous protective mechanism within cells that is responsible for monitoring mitochondrial mass to maintain mitochondrial balance. Will the negative effects caused by mtDNA instability be recognized and eliminated? Therefore, it is necessary to further elucidate the relationship between mitochondrial quality testing and ovarian aging.

### 2.3 Disorder of mitochondrial quality monitoring mechanism in ovarian aging

#### 2.3.1 Mutation of the key gene CLPP in mitochondrial unfolded protein response leads to depletion of ovarian reserve

Mitochondrial unfolded protein response (UPRmt), as a typical mitochondrial nuclear signal transduction process, promotes the high expression of nuclear-encoded mitochondrial stress proteins by transmitting mitochondrial damage signals to the nucleus (93, 94), helping to restore mitochondrial protein balance, thereby protecting electron transport chain complexes to avoid proteotoxicity. The maintenance of mitochondrial protein function, including correct folding, aggregation, and necessary degradation, requires the involvement of proteolytic enzymes and molecular chaperones. It is known that caseinolytic peptidase P(CLPP) is a highly conserved serine proteolytic enzyme and a key enzyme in UPRmt (95). Currently, it is reported that human *CLPP* gene mutation has a clinical phenotype of Perrault syndrome or POI (96, 97). In animal experiments, *CLPP*-/- mice showed auditory defects and complete infertility (98), and the ovarian reserve in mice showed an accelerated state of depletion with age (99, 100). The reason maybe that *CLPP*-/- mice activate the Sirolimus target (mTOR) pathway *in vivo (* 99), affecting the degradation of aggregated or misfolded cytochrome oxidase subunit 5A(COX5A) (97), blocking UPRmt, thus affecting the content and activity of complex IV in ETC, accumulating ROS and reducing mitochondrial membrane potential (MMP), and finally activating the internal apoptosis pathway. It follows that mutations in the key gene CLPP, where UPRmt is affected, will lead to ovarian reserve depletion in mice and humans. Therefore, we hypothesize that ovarian aging may be associated with differential expression of the CLPP gene, but providing a definitive conclusion is challenging due to the paucity of studies on ovarian aging-associated UPRmt. The inhibition of UPRmt process means a decrease in the genes driving mtDNA replication and transcription in the nucleus, and a limitation in the synthesis of new mitochondrial proteins.

#### 2.3.2 Obstacles in mitochondrial biogenesis in aging ovary

Mitochondrial biogenesis is a strict regulatory process that activates signaling molecules such as NRF1/2, TFAM, and TFBM through peroxisome proliferator-activated receptor-gamma coactivator-1alpha (PGC1α), driving the replication and transcription of mtDNA, translating it into proteins, and assembling it into new mitochondria (101). SIRT1/3 also activates PGC1α-NRF1 promotes mitochondrial synthesis (53, 54). When mitochondria sense the initiation of OS or UPRmt processes, mitochondrial biogenesis is activated to maintain mitochondrial balance. Current research found a significant downregulation of PGC1α expression in the ovaries of cyclophosphamide-induced injury in POI (54, 102). NRF1, SIRT1, and SIRT3 are all downregulated in the ovaries of reproductive aging (55–57). In the ovaries of SIRT3 gene knockout mice, there was a decrease in mitochondrial membrane potential, uneven distribution of mitochondria, and a decrease in mtDNA copy number (56). Ginsenoside Rg1 improves ovarian function by activating SIRT1 and coenzyme Q10 improves ovarian function by increasing the expression of SIRT1 in PGC1α in animal experiments (103, 104). In addition, Adenosine 5’-monophosphate-activated protein kinase (AMPK), which is associated with the onset of POI (105), can activate and interacts with PGC1α through various mechanisms (106). Research has found that exercise can increase intracellular energy metabolism, activate AMPK and PGC1α to increase intracellular calcium concentrations thereby increasing the induction of mitochondrial biogenesis through transcription factors (such as NRFs, TFAM) (101). Inhibition of the AMPK/SIRT3 pathway will induce mitochondrial protein hyperacetylation and mitochondrial dysfunction in pig oocyte (107). In summary, mitochondrial biogenesis is a regeneration process that maintains the number of mitochondria, replacing old and damaged mitochondria with new and healthy mitochondria. Mitochondria with decreased membrane potential will fuse with newly formed mitochondria (one aspect of mitochondrial dynamics), sharing an internal system to restore mitochondrial quality once again. However, the downregulation of key genes involved in mitochondrial biogenesis in aging ovaries may lead to obstacles in the regeneration process (55–57, 60).

#### 2.3.3 Defects in mitochondrial dynamics of aging oocyte

Mitochondria undergo continuous transformation through fusion and division states, which are manifested by morphological remodeling of the mitochondrial cristae and fracturing and lengthening of the tubular network, in order to achieve physiological functions. Mitochondrial fusion protein (MFN) mediate the assembly and fusion of mitochondrial inner and outer membranes to form a homogeneous mitochondrial network to maintain quality. Optic Atrophy 1(OPA1) is a GTPase that promotes fusion of the inner mitochondrial membrane and maintains cristae integrity. Dynamin-related protein 1 (DRP1), as core protein in mitochondrial division, can promote rapid changes in mitochondrial division activity according to cellular needs. Existing studies found that, the absence of MFN1 and MFN2 in mice oocyte resulted in infertility phenotype and loss of follicular reserve (108, 109), and the expression of DRP1 in aging oocyte was reduced (110). Low expression of MFN2 is associated with mitochondrial damage and apoptosis in ovarian tissue in POI model mice (111, 112). Since activated JNK phosphorylates MFN2, causing it to be degraded via the ubiquitin-proteasome system, the expression of MFN2 can be restored after supplementation with hydrogen sulfide or glutathione, antioxidants, or JNK inhibitors, and the improvement of mitochondrial morphology contributes to cell proliferation in ovarian cancer (113). In goat ovarian granulosa cell, Neuromedin S(NMS) treatment upregulates the expression of OPA1, MFN1, and MFN2 in the presence of NMUR2 knockdown, maintains mitochondrial fusion capacity and function, and thus regulates steroidogenesis (114). Therefore, we speculate that there are mitochondrial motility defects in aging oocyte. Due to the ability of mitochondrial fission to separate damaged mitochondria with low membrane potential from the entire mitochondrial network. Damaged mitochondria either fuse with newly formed mitochondria or are cleared through mitochondrial autophagy. The downregulation of mitochondrial fusion and fission ability implies an inevitable decrease in mitochondrial quality.

#### 2.3.4 Downregulation of mitochondrial autophagy in aging ovary

When mitochondrial damage cannot be repaired through fission/fusion, the role of mitochondrial autophagy becomes particularly important. Mitochondrial autophagy is achieved through the phagocytosis of damaged mitochondria by autophagosomes, primarily via the PINK1-Parkin pathway, which removes damaged and membrane potential loss mitochondria, and receptor-dependent mitochondrial autophagy such as BCL2/adenovirus E1B 19 kDa protein-interacting protein 3 (BNIP3), BCL2/E1B 19kDa interacting protein 3-like (BLIP3L or NIX), or FUN14 domain-containing protein 1(FUNDC1) (115). Studies have shown that maintaining mitochondrial autophagy is crucial for prolonging the reproductive capacity and oocyte quality of *C. elegans* and mice (116, 117). In particular, mitophagosome formation defects and accumulated damaged mitochondria were observed in germinal vesicle (GV) oocyte collected from the ovaries of older mice (118), which may be caused by PINK1 and Parkin proteins age-related accumulation and the degradation of ras-related protein rab-7a(RAB7) ubiquitination modification (118, 119). RAB7 is a monomeric guanosine triphosphate(GTP)-binding protein and an essential regulatory factor for the late endosomal/lysosomal network (120). Following treatment with RAB7 activators, the fertility of mice was improved. Therefore, Jin et al. suggested that excessive ubiquitination of RAB7 inhibits mitochondrial autophagy that should have been activated during ovarian aging (118). Another research reported a therapeutic strategy to ameliorate oocyte quality and reproductive outcome by enhancing mitophagy in aged mice (117). CoQ10 significantly increased PINK1 and Parkin proteins and improved embryonic development in postovulatory oocyte of pigs (104). From the above findings, it can be surmised that mitochondrial autophagy is down-regulated in senescent oocyte. Defects in the formation of mitochondrial phagosomes lead to the accumulation of damaged mitochondria and a decrease in mitochondrial quality. The decline in mitochondrial function ultimately leads to ovarian aging. Although the above results are exciting. It should be noted that so far, most of our understanding of mitochondrial autophagy has been through detecting the expression of proteins related to related pathways. However, for some reasons, such as the instantaneous occurrence of mitochondrial autophagy leading to cell apoptosis, it is challenging to measure mitochondrial autophagy accurately (121, 122).

**Figure 2:**
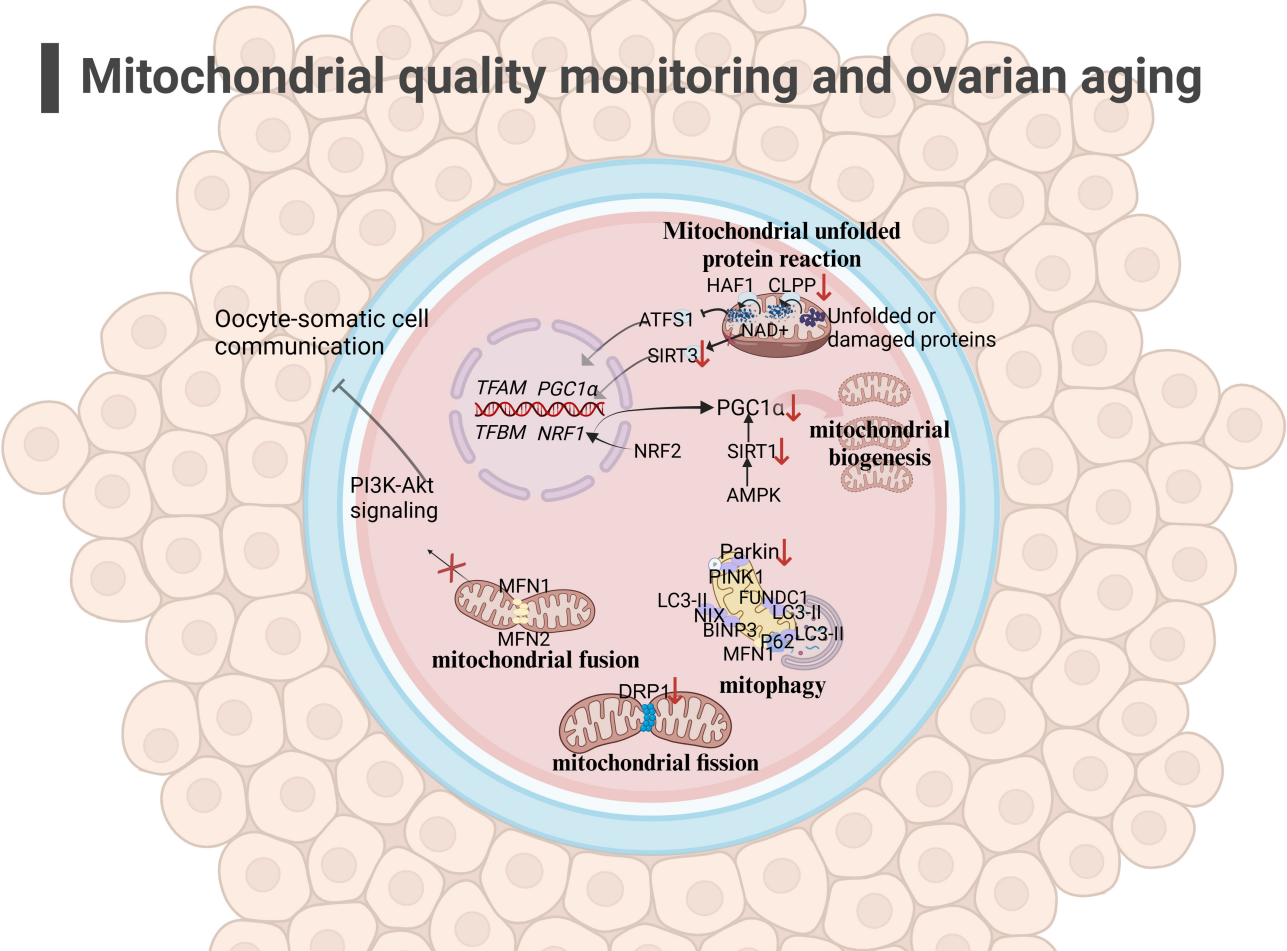
Mitochondrial quality monitoring and ovarian aging(the figure was created with BioRender.com) (1)mitochondrial fusion: The low expression of MFN1 ang MFN2 in the outer membrane in aging oocyte may affect mitochondrial fusion and inhibit the PI3K-AKT pathway, affecting oocyte-somatic cell communication (108, 109); (2) mitochondrial fission: The decreased expression of DRP1 in oocyte may affect mitochondrial function, leading to a decrease in oocyte quality (110); (3) mitophagy: Mitochondrial autophagy function decreases during ovarian aging (117, 118); (4) UPRmt: The decreased expression of CLPP and PGC1α in aging oocyte may hinder the occurrence of UPRmt (96, 97).

In summary, we have elucidated four aspects of mitochondrial quality monitoring that may be related to ovarian aging (as shown in **Figure 2**). Current researches indicate that mutations in the key gene CLPP of UPRmt can lead to depletion of ovarian reserves in both mice and humans. The expression of key genes involved in mitochondrial biogenesis, mitochondrial dynamics, and mitochondrial autophagy in aging ovaries has been downregulated. Therefore, various functions of mitochondrial quality monitoring in aging ovaries may have been downregulated to varying degrees. A decrease in mitochondrial quality usually means a decrease in mitochondrial cristae area and enzymes related to aerobic respiration in the matrix, resulting in insufficient mitochondrial energy supply and affecting the function of oocyte.

### 2.4 Mitochondrial dysfunction disrupts normal physiological function of oocyte

#### 2.4.1 Inadequate mitochondrial energy supply is a factor in meiotic errors

**Figure 3:**
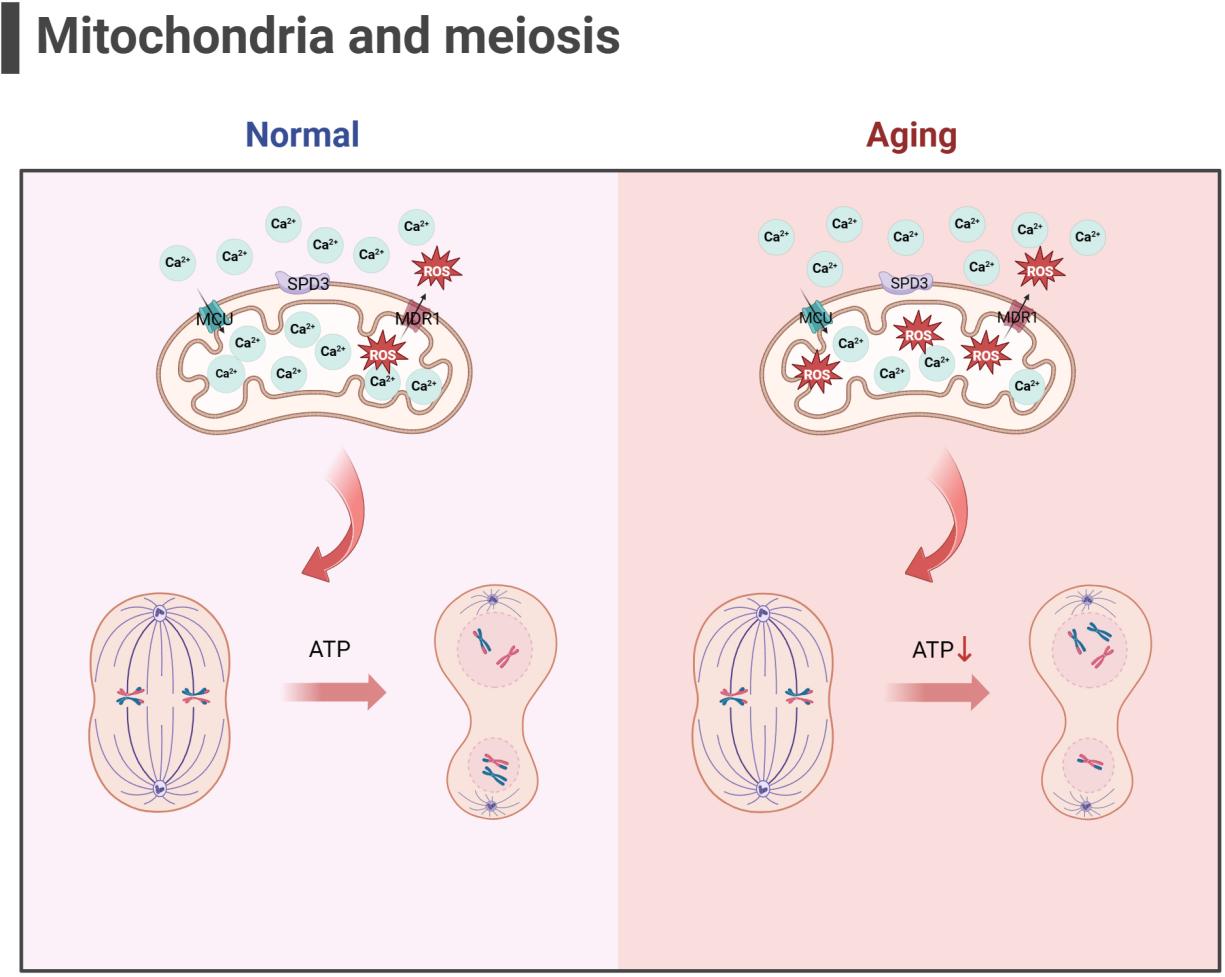
Mitochondria and meiosis in the oocyte (the figure was created with BioRender.com) MCU mediates the rapid entry of Ca^2+^ into mitochondria and provides high energy in an instant (129). MDR1 regulate ROS efflux from the inner mitochondrial membrane to maintain mitochondrial function (133). SPD3 may affect the abnormal pairing of homologous chromosomes which may be caused by the inhibition of mitochondrial function (132). Oxidative stress and mitochondrial ATP production disorders in aging oocyte may lead to abnormal spindle shape and increase chromosomal errors during meiosis in oocyte.

Some scholars have found that euploid oocyte screened from women with advanced age have implantation potential similar to that of young women with *in vitro* fertilization (IVF) (123). The production of aneuploid oocyte is the main reason for the decline in female fertility. The error of MI of female oocyte is considered to be the primary cause of human abortion and congenital defects (124, 125), and oocyte meiotic spindle morphology is a predictive marker of blastocyst ploidy (126), although mechanism of meiosis is complex. During meiosis, mitochondrial dysfunction significantly affects spindle assembly and ratio of kinetochore-microtubule connection (127, 128), and maternal age-related meiotic errors can be attenuated by reducing mitochondrial function (128). In the process of meiosis of mice oocyte, mitochondrial calcium uniporter protein (MCU) mediates the rapid entry of Ca2+ into mitochondria and provides high energy in an instant (129). The specific deletion of MCU leads to a low concentration of Ca2+ in mitochondria, low ATP levels, abnormal spindle assembly, and altered meiosis progression (129). Interestingly, the decrease of mitochondrial ATP concentration activates AMPK signal transduction. However, over-activated AMPK and Ca^2+^ overload result in OS, apoptosis, and meiotic cell cycle arrest and apoptosis in mammalian oocyte (130, 131). Above research demonstrated that precise mitochondrial Ca^2+^ homeostasis mediated by MCU is critical to oocyte meiosis. Moreover, the deletion of spindle defective protein 3 (SPD3) located on the outer membrane of mitochondria directly leads to the abnormal pairing of homologous chromosomes in the meiosis of *Caenorhabditis elegans*, which may be caused by the inhibition of mitochondrial function (132). Other proteins that regulate mitochondrial function, such as multidrug resistance protein 1 (MDR1) (133), regulate ROS efflux from the inner mitochondrial membrane, and RAB7 specific deletion regulates DRP1 phosphorylation (134), resulting in mitochondrial dysfunction, abnormal spindle shape, and increase of oocyte meiosis chromosome errors. In addition, SIRT3-/- mice aging oocyte exhibited spindle assembly interruption (55). These studies together highlighted the importance of mitochondrial energy supply for meiosis (as shown in **Figure 3**). In aging ovaries, the decline in mtDNA number and mutations, the down-regulation of mitochondrial quality monitoring mechanisms, and insufficient supply of mitochondria may persist, culminating in derangement of spindle assembly and motility, leading to an increase in meiotic chromosome errors in oocyte.

#### 2.4.2 Mitochondrial dysfunction disrupts the oocyte-cumulus cell crosstalk in aging ovaries

In the cumulus oocyte complex (COC), CCs continuously consume glucose to supply metabolic intermediates (such as pyruvic acid) to oocyte, which will be crucial to energy metabolism in the mitochondria of oocyte (135). CCs are GCs surrounding oocyte that participate in the reproductive and maturation processes of oocyte through intercellular communication. MFN1-/- mice oocyte showed DOR phenotype, as well as damaged communication between oocyte and CCs (cadherin and connexin were downregulated), which may be caused by mitochondrial dysfunction and mitochondrial dynamics changes, the accumulation of ceramide in oocyte and the damage of PI3K-AKT signal transduction (109, 136). Ceramide is considered an important inducer of programmed cell death, and ceramide metabolic enzyme therapy can improve the quality of oocyte and embryos and the outcome of IVF (137). The PI3K-AKT pathway is an important signaling pathway for FSH to regulate glucose uptake in GCs and prevent ovarian aging (138). In addition, mitochondrial dysfunction of MII oocyte in AMPK -/- mice increased abnormally, PGC1α level decreased, ATP concentration decreased from normal, and connexin 37 and n-cadherin, which are involved in connection and communication between oocyte and CCs, were downregulated (139). The emergence of mitochondria-associated gene mutant mice with down-regulated expression of junctional gap junction proteins may have affected the delivery of some small molecules from the colobus cells to the oocyte, which in turn affected the quality of the oocyte. The above research provides evidence for mitochondrial dysfunction leading to abnormal communication between oocyte and CCs, supporting the theory of mitochondrial dysfunction leading to ovarian aging. However, there is a paucity of relevant studies, which need to be further clarified by rigorous experiments.

#### 2.4.3 Hyperactivation of the mitochondrial apoptotic pathway is present during ovarian aging

**Figure 4:**
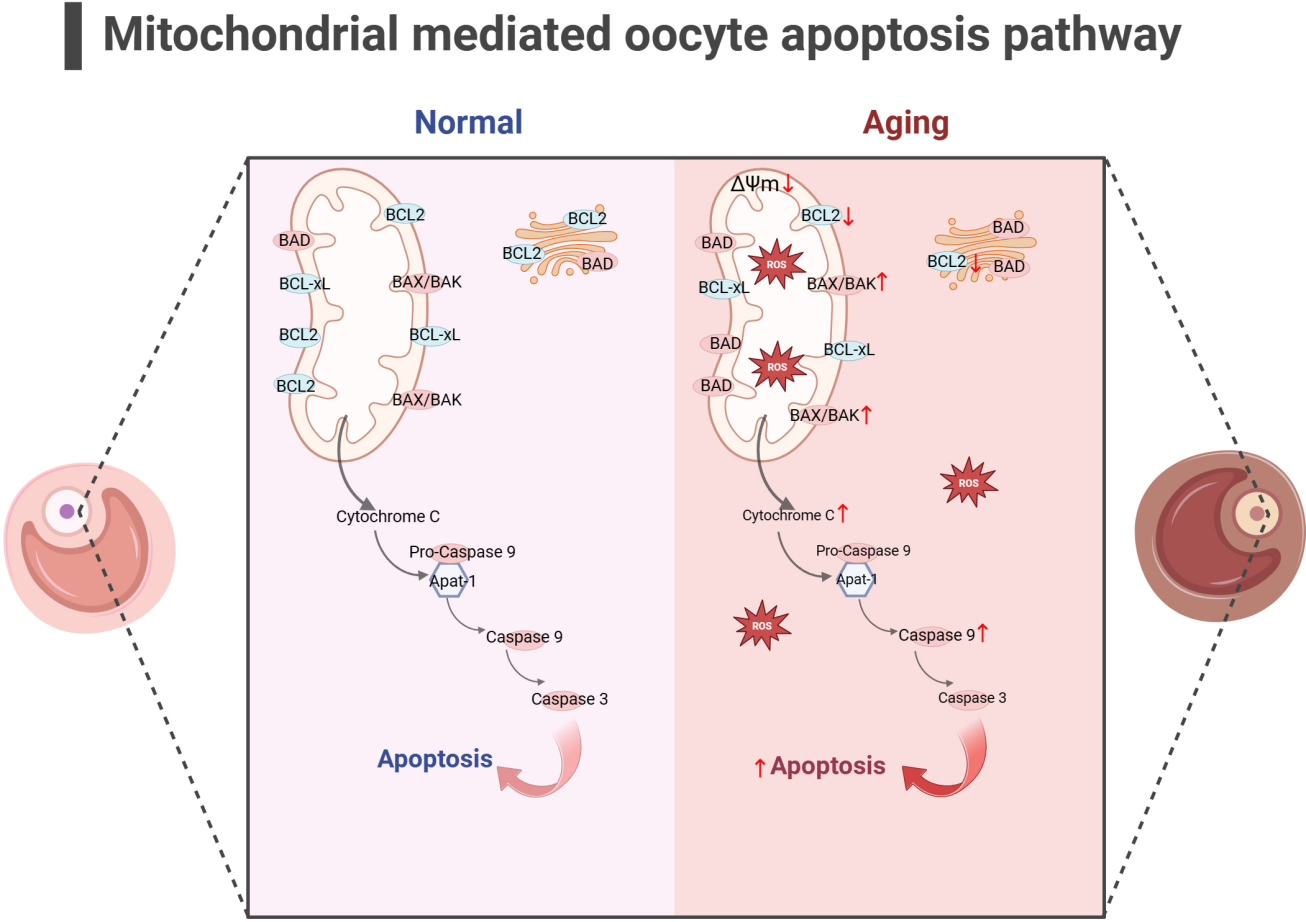
Mitochondrial-mediated oocyte apoptosis pathway (the figure was created with BioRender.com) In aging oocyte, excessive cell apoptosis signals are activated under stimuli such as mitochondrial oxidative stress and decreased mitochondrial membrane potential. At the same time, the expression of anti-apoptotic proteins such as BCL2 decreased, while the expression of apoptotic proteins such as BAK, CytC and Caspase9 increased (140–142).

In addition to energy generation, the important role of mitochondria in cells is also reflected in regulating cell apoptosis. Mitochondria is the central organelle of the apoptosis pathway, having an irreplaceable role in apoptosis. Under physiological conditions, Cytochrome c (Cytc) is the carrier for transferring electrons in ETC, establishing the mitochondrial transmembrane potential and generating ATP. BCL2 and BCL-xL form heterodimers with BCL2-associated X(BAX) and BCL2 homologous antagonist/killer (BAK), maintaining the integrity of the mitochondrial outer membrane and preventing mitochondrial apoptosis response. When stimulated by apoptosis, BAX/BAK forms an oligomer complex and inserts into the outer membrane pores of mitochondria, leading to changes in mitochondrial osmotic pressure, abnormal activation of Cytc release channels, and initiation of downstream caspase cascade reaction to induce apoptosis. Consistent with this, many studies have illustrated upregulated expression of apoptotic protein BAX, Cytc and Caspase 9 in the ovaries of mice with POF and POI animals (140–142), while the expression of anti-apoptotic protein BCL2 was downregulated. After intervention, the expression of these proteins was reversed, partially restoring ovarian function. Gene knockdown experiments have shown that BAX mutations prolong the fertility of female mice and alleviate health issues related to aging (143, 144). In addition, mitochondrial dysfunction and reduced ATP production may disrupt the normal physiological functions of cells (145, 146), leading to cell apoptosis. In summary, mitochondrial dysfunction can disrupt cellular antioxidant capacity and disrupt the OXPHOS process, hindering ATP production. Mitochondria themselves can perceive a decrease in membrane potential, sense apoptotic signals, and initiate the mitochondrial apoptotic pathway. The excessive activation of mitochondrial apoptosis pathway is likely closely related to the occurrence of ovarian aging (as shown in **Figure 4**). Because there is excessive apoptosis of GCs and increased follicular atresia during ovarian aging.

#### 2.4.4 The link between mitochondrial dysfunction and telomere damage as one of the latest mechanisms of ovarian aging

**Figure 5:**
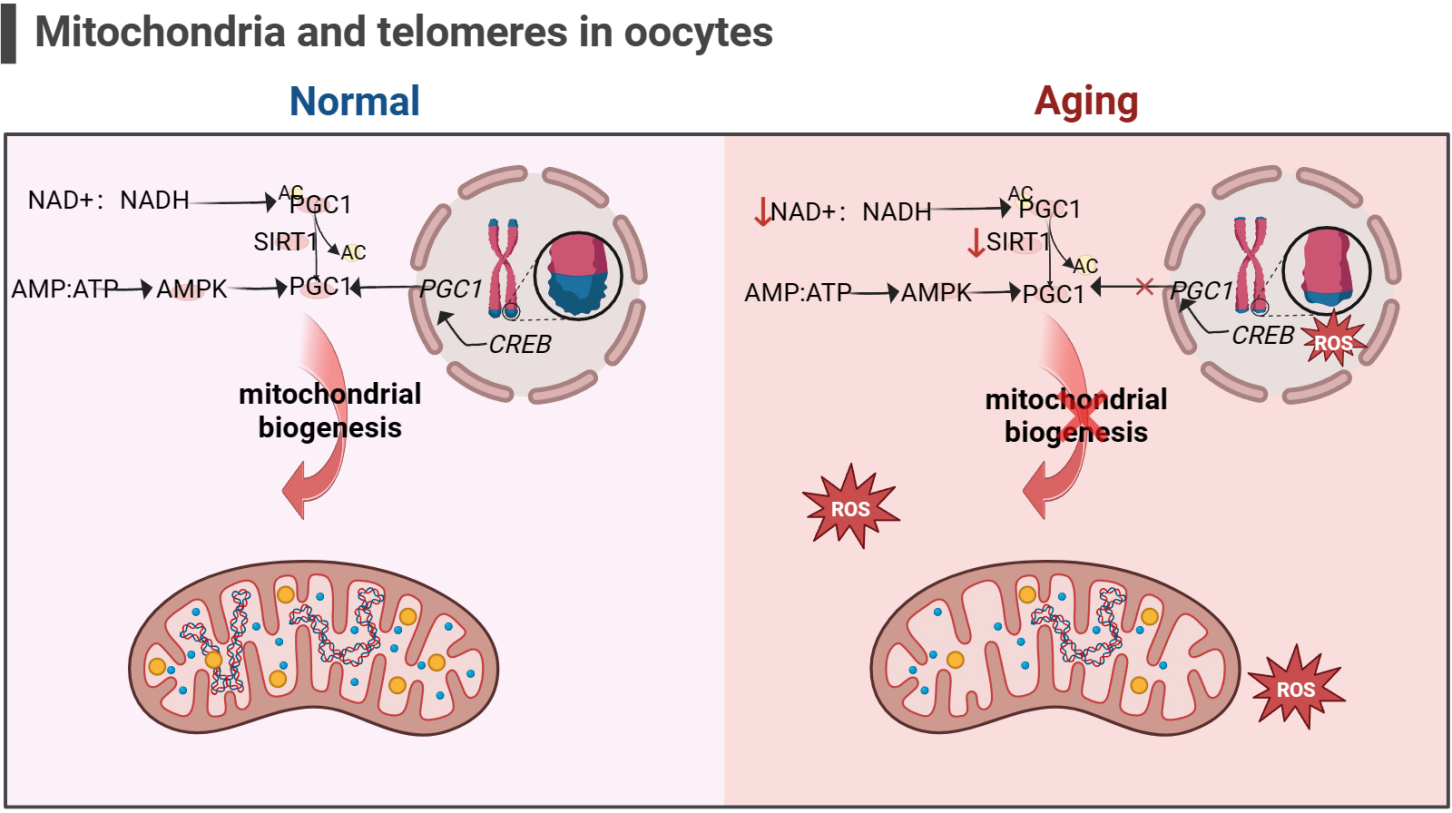
Mitochondria and telomeres in the oocyte (the figure was created with BioRender.com) The cAMP response element-binding protein (CREB) is a co-factor for PGC1 expression. Abnormal telomere function can activate p53, thereby inhibiting the expression of PGC1. The decrease in PGC1 expression in turn inhibits mitochondrial biogenesis, leading to a decrease in the ability of cells to resist oxidative stress (156). In addition, increasement in the proportion of intracellular AMP/ATP and NAD+/NADH also promotes PGC1 expression, but this is not suitable for aging cells, as the content of NAD+ and SIRT1 significantly decreases (53, 54).

In addition, the telomere theory is one of the latest mechanisms to explain female reproductive aging (147).Telomeres are nucleoprotein complexes at the end of chromosomes, maintaining the integrity of chromosomes and inhibit DNA damage reactions at the free end of chromosomes (148). It is widely recognized that telomeres are susceptible to oxidative damage, and OS markers have been shown to exhibit a negative correlation with telomere length (149). Due to the significant features of ovarian aging, such as reduced ATP production and increased ROS accumulation (88), telomere loss and telomere-driven replicative senescence caused by OS may be important mechanisms of ovarian aging (150). In addition, there is a link between telomere function and mitochondrial biosynthesis. Existing studies have found decreased telomerase activity in ovarian GCs of POI patients (151, 152). The lack of telomerase activates p53 which in turn binds and represses PGC1α and PGC1β promoters, thereby inhibiting mitochondrial biogenesis (153, 154). The dysfunction of mitochondrial biosynthesis leads to the decline of cell resistance to OS and further accelerates the modification of 8-hydroxy deoxyguanosine on the telomere guanosine base. In addition, the link between telomere shortening and mitochondria is also mediated by NAD+-SIRT1-PGC1α axis establishment (124, 155, 156). The DNA repair process after telomere damage consumes NAD+, and the loss of SIRT1 activity will further affect the mitochondrial biogenesis mediated by PGC1α (155). In aging ovaries, the decrease in SIRT1 expression is a common phenomenon (55). Thus, the excessive occurrence of mitochondrial OS may accelerate telomere shortening at the ends of chromosomes, which in turn can inhibit the expression of the key gene for mitochondrial biosynthesis, PGC1α, through the activation of P53, leading to a decline in intracellular mitochondrial function, which further affects the DNA repair process. Ultimately, this vicious cycle of the above leads to the onset of ovarian senescence (as shown in **Figure 5**). Given the current research results, it is difficult to explain the sequence between telomere damage and mitochondrial dysfunction. Still, the two seem to interact with each other to lead to ovarian aging in women.

#### 2.4.5 Epigenetic regulation involving mitochondria influences oocyte senescence

Oocyte quality decline during ovarian aging occurs in part through epigenetic regulation. Intermediates generated by metabolic processes within mitochondria can generation and modify nuclear epigenetic marks to achieve important mediators of mitochondrial-nuclear communication (157). Histone methyltransferases (HMTs) and histone demethylases (HDMs) are responsible for histone methylation status. S-adenosyl methionine (SAM) produced by the cytoplasmic methionine homocysteine cycle in the mitochondrial folate cycle is a donor of histone methyltransferases (HMTs) (158). Besides, the acetyl-CoA-producing enzyme ATP-citrate lyase (ACL) also regulates DNA methyltransferase 1 (DNMTl) (159), which affects the level of DNA methylation. Pyruvate, ketones, amino acids, citrate, acetate, and beta oxidation of lipids can produce acetyl-CoA, which is the substrate for the histone acetyltransferases (HAT) (160). Under conditions of energy and acetyl-CoA enrichment, this leads to histone acetylation and gene transcription. Histone demethylases (HDMs) comprise two major classes: lysine-specific demethylases (LSD) and Jumonji C domain demethylases (JMJD). LSD1 catalyzes the demethylation of mono- or dimethylated H3K4 and H3K9, whereas LSDs act as receptors in the mitochondrial ETC (161). JMJD-mediated demethylation of histones requires α-ketoglutarate (162), a substrate produced mainly by the tricarboxylic acid (TCA) cycle in the mitochondrial matrix. Current research has found that low methylation of DNMT1/DNMT3a/DNMT3b/DNMT3L promotes oocyte aging (17). Specific disruption of LSD1 led to a significant increase in autophagy through its H3K4me2 demethylase activity and a decrease in the number of oocyte in perinatal mice, leading to depletion of oocyte (163). Epigenetic enzymes recognize, add, and remove epigenetic markers on DNA and histones. Due to the association between the formation of epigenetic enzymes and mitochondrial metabolism, the transmission of mitochondrial metabolite levels or stress signals may lead to various epigenetic changes. Unfortunately, few studies have directly explored the association between mitochondrial epigenetic regulation and ovarian aging.

In summary, mitochondrial dysfunction during cellular aging may lead to spindle assembly and motility during meiosis by affecting energy supply. Both MFN1-/- and AMPK -/- mice showed mitochondrial dysfunction and abnormal communication between oocyte and GCs. In addition, the occurrence of mitochondrial dysfunction may directly initiate the mitochondrial apoptosis pathway, leading to follicular atresia. Excessive OS during aging not only activates the mitochondrial apoptosis pathway, but may also accelerate telomere shortening. Telomere shortening hinders the recovery of mitochondrial function by inhibiting mitochondrial biosynthesis. Finally, mitochondrial metabolites may also affect the expression of epigenetic enzymes and promote ovarian aging.

## 3 Methods to slow down ovarian aging and prolong reproductive lifespan by intervening in mitochondrial function

At present, there is no effective technology in clinical practice to prevent the occurrence of female reproductive aging, and the current treatment methods are still in the exploratory stage. However, it is worth celebrating that some animal experiments have shown great potential in improving female reproductive function. Below we will discuss each of the currently discovered therapeutic mechanisms of drugs for mitochondrial damage.

### 3.1 Mitochondrial nutrition therapy

We summarize the therapeutic drugs targeting mitochondria to prevent ovarian aging in **Table 1** and classify them according to mitochondrial energy metabolism, quality control, and mitochondrial apoptosis pathways.

**Table 1:** Drugs that improve mitochondrial function.

Classification	Drug	Mechanism	References
improve mitochondrial energy metabolism	coenzyme Q10, vitamin E, vitamin C, astaxanthin,L-carnitine, melatonin, quercetin, resveratrol, metformin, ginsenoside Rb1	reduce superoxide radicals and scavenge hydrogen peroxide	(40, 44, 164–171)
	ginsenoside Rb1	Akt-FOXO1 interaction	(44)
	metformin	regulation of calcium ion homeostasis	(40)
	NAD+ nucleoside	promotes TCA cycle	(172, 173)
coordinate mitochondrial quality control	ginsenoside Rg1, coenzyme Q10,	SIRT1 or PGC1α activator	(103, 104)
	melatonin, metformin	AMPK activator	(104, 174)
	resveratrol, coenzyme Q10	upregulate Parkin-induced mitochondrial autophagy	(104, 175)
	NAD+ nucleoside	mitochondrial autophagy enhancers	(41)
regulating mitochondrial apoptosis pathway	metformin	inhibit release of BAD and caspase	(40)
	resveratrol	inhibition of caspase 3 and BAX, upregulation of BCL2	(176)

#### 3.1.1 Drugs for improving mitochondrial energy metabolism

Research has shown that multiple antioxidants can restore mitochondrial function by reducing ROS production and stimulating antioxidant production. It mainly includes coenzyme Q10 (177), vitamin E (164), vitamin C (164), L-carnitine (165, 166), melatonin (167, 168), quercetin (169), resveratrol (170), astaxanthin (171), and ginsenoside Rb1 (44).

In addition, ginsenoside Rb1 also promotes Akt binding to FOXO1 and inhibits OS occurrence (44). Metformin regulates calcium homeostasis to prevent follicular atresia of aging ovaries of Laying Chickens (40). NAD+/NADH, or coenzyme I, is a redox cofactor and enzyme substrate in mitochondria, critical for energy metabolism, DNA repair, and epigenetic regulation in cells. In the ovaries, the level of NAD+ decreases with age in a dependent manner (172, 173). After supplementing NAD+ precursors (NR or NMN), ovarian function in elderly mice was restored, and the mitochondrial TCA cycle was improved at the micro level (172, 173).

#### 3.1.2 Drugs for coordinating mitochondrial quality control

Normal mitochondrial function and internal environmental homeostasis require close coordination between mitochondrial biogenesis and clearance. Compounds coordinating mitochondrial quality control are expected to become effective therapeutic interventions for ovarian aging. It is reported that ginsenoside Rg1 can increase SOD, CAT levels, and activate SIRT1 in POF mice model (103). CoQ10 significantly prevented aging-induced oxidative stress, and increased mitochondrial biogenesis (SIRT1 and PGC1α) and mitophagy (PINK1 and Parkin)-related proteins in postovulatory oocyte of pigs (104). Resveratrol can increase the expression of PINK1 and Parkin proteins regulate the autophagy ability of mitochondria, and protect ovarian function (175). Metformin and Melatonin are AMPK activators (105, 174), which could increase intracellular calcium concentrations thereby increasing the induction of mitochondrial biogenesis through transcription factors (such as NRFs, TFAM) (111). After 20 weeks of treatment with NMN in 40-week-old mice (41), mitochondrial biogenesis, autophagy level and protease activity in ovarian GCs were increased, and the ovarian reserve was rescued to some extent.

#### 3.1.3 Drugs that regulate mitochondrial apoptosis pathways

The study found that Resveratrol treatment reduced the expression of mitochondrial apoptosis promoting protein caspase 3 and BAX and upregulated BCL2 expression in POI model mice (176). Metformin can inhibit the release of mitochondrial apoptosis factors (BAD and caspase) in Laying Chickens (40). However, whether these benefits observed in animal models can be replicated in humans remains to be determined.

Currently, various potential drugs targeting mitochondria have been developed (**Table 1**). However, these drugs are not necessarily suitable for preventing ovarian aging. Various drugs have been found to have positive effects in resisting OS, improving OXPHOS efficiency, promoting mitochondrial occurrence and autophagy, and inhibiting mitochondrial apoptosis in animal experiments. Whether these benefits observed in animal models can be replicated in the human body remains to be determined.

### 3.2 Mitochondrial replacement therapy

Mitochondrial transplantation was initially primarily used to prevent disease transmission caused by mtDNA mutations, becoming a viable alternative to avoiding damaged mitochondrial offspring inheritance. Subsequently, due to the increasingly important role of mitochondria in human reproduction, people began to think about improving the quality of gametes by enhancing the quality of mitochondria.

#### 3.2.1 Autologous mitochondrial transplantation technology

The autologous mitochondrial transplantation technology (178), focuses on increasing the number of healthy mitochondria in cells while avoiding the introduction of third-party DNA in gametes. It is reported that injection of mitochondrial from oogonial precursor cells(OPCs) while intracytoplasmic sperm injection (ICSI) can improve the quality of oocyte and pregnancy rate in the ICSI cycle of women with multiple IVF failure (70, 71). Unfortunately, a more rigorous tri-blinded, randomized, and single center-controlled study conducted in Spain in 2019 found that mitochondrial transfer of OPCs did not improve embryonic development potential and pregnancy rate in patients with previously IVF failed (179). Recently, it has been confirmed that autologous adipose stem cells (ASCs) mitochondrial transplantation can improve the quality of oocyte in juvenile or aged mice (180–182), while Sheng et al. found negative results (183). It should be noted that the mitochondrial dysfunction of aging oocyte seems to have occurred long ago. Most of the common aneuploid oocyte originate from MI (126, 184). It may be too late to save the quality of MII oocyte by ICSI injection of active mitochondria. Furthermore, the origin of mitochondria is an issue, and whether oogonial stem cells exist and how to obtain them is a highly controversial issue (185, 186). In summary, the efficacy of the above mitochondrial transplantation methods in improving ovarian function is controversial, and they are not considered standard treatments.

Tang et al. have established a noninvasive optimized autologous mitochondrial transplantation technique: inducing mice autologous umbilical cord mesenchymal stem cells into GCs (iGCs) and co-cultured them with weakened zona pellucida GV in growth differentiation factor 9 (GDF9) containing medium for three days. Tang et al. have found that mitochondria migrate from iGCs to GV oocyte through transzonal filopodia, significantly improving these oocyte’ maturation rate, quality, and developmental potential (187). The birth rate of aged mice after embryo transfer has also been improved (187). In **Table 2**, we summarized the mitochondrial transplantation methods of from different sources. Due to its non-invasive and early intervention characteristics, it is worth further research. In the future, we look forward to researching and solving the source of mitochondrial acquisition, achieving timely and non-invasive transplantation as much as possible, and providing promising strategies for improving the quality of oocyte and the fertility of women with advanced age.

**Table 2:** Comparison of mitochondrial transplantation methods from different sources.

Mitochondrial source	Model	Advantage	Disadvantage	Reference
oogonial precursor cells	human	high homology; mitochondria derived from stem cells	difficulty in obtaining invasive procedures; mitochondrial transplantation during Metaphase II	(70, 71, 179)
adipose-derived stem cells	mice	Rich sources; easy to obtain; low immune rejection	mitochondrial transplantation during Metaphase II	(180–183)
umbilical cord-derived mesenchymal stem cells	mice	non-invasive procedures; mitochondrial transplantation during germinal vesicle	differentiation needs to be induced *in vitro*	(187)

#### 3.2.2 Cytoplasmic transfer and germline nuclear transfer

A study in 1998 reported that Cohen et al. injected a small amount of oocyte cytoplasm directly from the donor oocyte into the oocyte of patients with recurrent implantation failure (RIF) through cytoplasmic transfer (CT), and successfully achieved live birth (188). Unfortunately, specific abnormalities (chronic migraine headaches, mild asthma, minor vision and minor skin problems et al.) were found in the investigation of the health status of offspring, and the safety and benefits of CT are still unclear (189), although it is uncertain whether CT causes these abnormalities. However, there are concerns about mtDNA heterogeneity in CT (190), and currently, the US FDA has suspended this study.

Previous nuclear transfer (NT) is used clinically in patients with mtDNA diseases to prevent the transmission of maternal mutated mtDNA to the next generation. The new NT technology can improve embryonic development by transferring nuclear DNA from oocyte with inferior cytoplasm to oocyte with higher fertility potential. NT includes spindle transfer (ST), pronuclear transfer (PNT), and polar body transfer (PBT).

ST refers to the transfer of spindle apparatus from MII oocyte to denucleated MII donor oocyte (191). A pilot study conducted in Europe reported the feasibility of ST technology, targeting 25 couples with recurrent IVF failures and giving birth to 6 newborns over 28 ST and ICSI cycles (192). However, it is worth noting that one child with the same low mtDNA carryover (0.8%) in the blastocyst stage showed an increase in maternal mtDNA haplotype, accounting for 30% to 60% of the total number at birth (192). The mtDNA haplotype phenomenon may be due to the specific interaction between nuclear and mitochondrial coding genes. Heterogeneous mice model experiment show that one of the mtDNA haplotypes gradually dominates during oogenesis and early embryonic development (193). Therefore, before applying NT to clinical practice, it is necessary to carefully consider the occurrence of mtDNA mutations in patients.

In addition, PNT is a nucleoplasmic replacement technique, which refers to the process of removing the male and female protoplasts together and transplanting them into a new cytoplasm after the oocyte is fertilized. It was found that by replacing the cytoplasm of young mice, the MMP of oocyte from ST or PNT-reconstructed aged mice was increased, the probability of spindle and chromosome misalignment was decreased, and the rate of embryo haploidy and blastocyst formation was improved (194, 195). Several human preclinical studies have also reported the feasibility of the PNT technique in human zygotes (196), where early PNT performed 8 hours after ICSI can achieve blastocyst formation rates at control levels.

PB1T and PB2T also emerged as a strategy to prevent the spread of harmful mtDNA mutations. Unfortunately, the rate of reconstructed PB1T oocyte developing from zygotes to the blastocyst stage (42%) was lower compared to the control (75%) (197). Subsequently, researchers optimized the use of PB2T in human oocyte through new technologies. They found that PB2T embryos produced by *in vitro* maturation (IVM) oocyte exhibited similar development and diploid rates to the ICSI control group (198). It is important to note that more studies are needed to improve the efficiency and safety of the PNT technique and to provide at least evidence of live birth before it can be considered for clinical use.

In **Table 3**, we summarized the research methods for cytoplasmic transfer and germline nuclear transfer. It is worth noting that CT and NT cannot correct fertility disorders in all women with advanced age by improving receptor mitochondrial function, and the use of PGT to evaluate embryo ploidy remains an important choice. Because if there are genetic abnormalities in the spindle apparatus or polar body of women with advanced age, CT and NT are of little value. In 2015, the UK became the first country to approve the use of mitochondrial donations. More research evaluating the safety of this technology for women with low fertility is warranted.

**Table 3:** Comparison of cytoplasmic transfer and germline nuclear transfer.

Technique	Model	Results	Disadvantage	Reference
cytoplasmic transfer	human	successfully achieved live birth in patients with recurrent implantation failure	concerns about the health status of offspring and mtDNA heterogeneity	(189)
spindle transfer	human	6 newborns in 28 cycles	an increase in maternal mtDNA haplotype	(192)
pronuclear transfer	mice	increased the MMP of oocyte, the rate of embryo haploidy and blastocyst formation; decreased the probability of spindle and chromosome misalignment	species diversity; inability to correct aneuploidies already present in some aged MII oocyte	(194, 195)
pronuclear transfer	human	early PNT performed 8 hours after ICSI can achieve blastocyst formation rates at control levels	unreported pregnancy outcomes	(196)
first polar body transfer	human	the rate of blastocyst (42%) was lower compared to the control (75%)	unreported pregnancy outcomes	(197)
second polar body transfer	human	similar development and diploid rates in IVM compared to ICSI control	unreported pregnancy outcomes	(198)

### 3.3 Biomaterials and advanced technologies for preventing ovarian aging

Over the years, various strategies have been developed to maintain women’s Fertility. Recently, advances in biomaterials and technology have shown the potential to prevent ovarian aging. Exosomes from various sources, such as human umbilical cord mesenchymal stem cell-derived exosomes (hUCMSC-exos) (199), human amniotic mesenchymal stem cell-derived exosomes (hAMSC-exos) (200), human amniotic epithelial cell-derived mitochondria (hAEC-exos) (201), human amniotic fluid mesenchymal stem cells (AFMSC-exos) can decreased the ROS levels (202), increase the expression of anti-apoptotic genes (such as BAD and BCL2) and reduce the expression of pro-apoptotic genes (such as CPP32 and BAX) by transferring functional miRNAs (such as miR-320a, miR-1246, and miR-21), thereby inhibiting the mitochondrial apoptotic pathway and preventing ovarian GCs apoptosis in POI mice and primitive follicle activation. In mice ovarian experiments, transplantation of platelet-rich fibrin scaffolds indirectly reduced OS, thereby improving ovarian endocrine function and follicle formation (203). The effect of biomaterials on improving ovarian function is mainly based on the results of animal experiments. However, it is necessary to clarify the effectiveness of biomaterials in evaluating and treating ovarian aging, and to use effective preclinical models to accurately predict the therapeutic outcomes of these biomaterials.

In summary, some potential therapeutic drugs or technologies for improving mitochondrial quality have been developed or clinically tested. However, no treatment method is definitely suitable for preventing ovarian aging. Due to the highly complex process of reproduction, some treatment methods that have been introduced into clinical practice need to undergo comprehensive evaluation and be determined to have no side effects before being applied in clinical practice.

## 4 Focus on mitochondria of human oocyte during assisted reproduction technique

Age related infertility is mainly related to a decrease in the quantity and quality of oocyte, which limits a woman’s ability to conceive. Currently, an increasing number of women worldwide are seeking help from ART for conception. The need between mitochondria, infertility, and ovarian aging has attracted people’s attention. There is a close relationship between mitochondria and the declining quality of oocyte with age. The instability of mtDNA leads to the accumulation of mtDNA mutations in oocyte, posing a risk of transmitting mitochondrial abnormalities to offspring. Overactivation of OS during ovarian aging disrupts OXPHOS, induces telomere shortening, and stimulates cell apoptosis. However, due to the decrease in mtDNA copy number and the downregulation of mitochondrial quality monitoring ability, mitochondrial homeostasis is difficult to recover. At the cellular level, the mitochondrial apoptosis pathway is overactivated and ATP required for meiosis is lacking. **Table 4** summarizes and highlights the findings and links between mitochondrial dysfunction and infertility. During the oocyte developmental maturation stage, there is a process of transmission of damaged mitochondria to the embryo. Studies have shown that abnormalities in mitochondrial structure and function may be responsible for abnormal embryonic development in ART cycles.

**Table 4:** Link between mitochondrial damage and infertility.

Mechanism	Species	Targets	Results	References
changes in mitochondrial genome	human	TFAM	a recessive variant in TFAM causes mtDNA depletion associated with POI	(59)
	human	TFAM, POLG	a decreased mtDNA content in DOR, and a positively correlation between the quality of oocyte with the expression of TFAM and POLG in cumulus cells	(60)
	human	TFAM	women with moderate TFAM expression in follicular fluid granulosa cells showed better IVF outcomes	(66)
	human		the mtDNA copy number in cumulus cells can positively predict embryo quality and developmental outcome in IVF	(204–207)
	human		the mtDNA copy number in granulosa cells was significantly negatively correlated with age in the POSEIDON low prognosis groups	(67)
	human		a significantly lower mtDNA copy number in unfertilized oocyte and uncleaved embryos in women >40 years age	(35)
	human	POLG	mutations in POLG cause premature aging	(208)
	human	POLG	POLG is associated with female menopause	(89)
	human		there was no correlation between mtDNA deletion, rearrangement, and mutation of human oocyte and maternal age	(75, 77, 79, 80)
oxidative stress	mice, human	IDH1, JNK, p38 MAPK	ROS inhibits proliferation and promotes apoptosis in granulosa cells	(48)
	rat,human	SIAH1,TRF2	ROS promotes telomere shortening and granulosa cell aging	(49)
	human	H202, HIF-1α,VEGF	H_2_O_2_ stimulated oxidative injury and apoptosis in GCs	(50)
	human	SOD, CAT, GSHPx	decreased expression of antioxidant enzymes in follicular fluid of women with advanced age	(44, 45)
	mice	SIRT1	SIRT1 signaling protects mice oocyte against oxidative stress and is deregulated during aging	(53)
	mice	PRX2, JNK	ROS accelerates ovarian failure	(51)
	mice, human		an increase in ROS and a decrease in MMP may lead to spindle and chromosomal abnormalities in aging oocyte	(42)
	human		the assessment of the oxidative stress rate may be helpful in evaluating *in vitro* fertilization potential	(43)
mitochondrial dynamics	mice	MFN1, MFN2	lack of MFN1 and MFN2 in oocyte resulted in accelerated follicular depletion and impaired oocyte quality	(108)
	mice	MFN1	absence of MFN1 and resulting apoptotic cell loss caused depletion of ovarian follicular reserve	(109, 136)
	mice	MFN2	low expression of MFN2 was associated with mitochondrial damage and apoptosis of ovarian tissues in the POI mice	(111)
	human	MFN2	MFN2 expression was remarkably lower in granulosa cells from the DOR patients and decreased as age increased	(112)
	mice	DRP1	DRP1 deficiency affected follicle maturation and ovulation	(110)
mitochondrial biogenesis	mice	PGC1α	the expression of PGC1 in the ovaries of POI mice induced by cyclophosphamide was significantly downregulated, and mitochondrial damage occurred	(54, 102)
	mice	SIRT1	SIRT1 deficiency led to a decrease in the number of oocyte and premature infertility	(55)
	mice	SIRT3	SIRT3 deficiency accelerated ovarian reserve depletion	(56)
	human	SIRT3	decreased SIRT3 mRNA in granulosa cells of DOR and women with advanced age	(57)
mitochondrial autophagy	Caenorhabditis elegans	PINK1	the absence of PINK1 shortened the reproductive span of C.elegans	(116)
	mice		polyamine metabolite spermidine restored oocyte quality by enhancing mitochondrial autophagy in elderly female mice	(117)
	mice	RAB7, PINK1, PRKN	the regulation of mitophagy affected oocyte meiosis and oocyte quality control during ovarian aging	(118)
mitochondrial unfolded protein response	human	CLPP	mutations in CLPP cause Perrault syndrome and POI	(96, 97)
	mice	CLPP	mutations in CLPP cause complete sterility	(98)
	mice	CLPP, mTOR, COX5A	CLPP deficiency accelerated the depletion of ovarian follicle reserves	(99, 100)
mitochondrial apoptotic	mice, rat	BAX, CytC, Caspase9, BCL2	excessive occurrence of mitochondrial apoptosis in POF and POI	(140)
	mice	BAX	absence of BAX protein extends fertility and alleviates age-related health complications	(143, 144)
mitochondrial dysfunction and telomere damage	human		reduced telomerase activity in granulosa cells of POI patients	(151, 152)
Inadequate mitochondrial energy supply and meiotic errors	mice	MCU	oocyte of MCU knockdown failed to correctly assemble the spindle during meiosis	(129)
	Caenorhabditis elegans	SPD3	the absence of SPD3 can lead to homologous chromosome pairing defects	(132)
	mice	MDR1	MDR1 mutation leads to abnormal meiosis and decreased oocyte quality	(133)
	mice	RAB7, DRP1	RAB7 GTPase regulates actin dynamics for DRP1-mediated mitochondria function and spindle migration in oocyte meiosis	(134)
mitochondrial dysfunction and abnormal intercellular communication	mice	AMPK	lack of AMPK can alter oocyte quality through energy processes and oocyte-somatic communication	(139)

### 4.1 How do abnormal mitochondria in aging oocyte affect ART outcomes?

#### 4.1.1 The mtDNA content of oocytes and cumulus cells affects embryo quality

Prior to oocyte maturation, the mitochondria of the oocyte is virtually quiescent to prevent genetic mtDNA mutations, and the energy to support oocyte maturation is supplied primarily by the surrounding CCs and GCs. This observation is reflected in the fact that the number of oocyte obtained from *in vitro* fertilization (IVF), basal follicle-stimulating hormone (FSH) levels, and anti-Mullerian hormone (AMH) are the determining factors of mtDNA content (209), and the copy number of mtDNA in CCs can positively predict embryo quality and developmental outcomes (204–207). In addition, a significant reduction in mtDNA copy number was found in unfertilized oocyte from women with advanced age, and there was a significant positive correlation between cytoplasmic volume of the cleavage sphere of uncleaved embryos and mtDNA copy number (35). In addition, oocyte from women with advanced age had higher mtDNA copy numbers after IVM than younger women, which may be related to spindle abnormalities and increased oxidative stress in IVM (210).

It has been suggested that the survival rate of early mammalian embryos is related to their quiet metabolism (211). Research indicates that the mitochondrial oxygen consumption rate of morula embryos is linked to maternal age rather than mtDNA content (212, 213). However, by the blastocyst stage, as differentiation occurs within the embryo, mtDNA replication rapidly recovers, and the TCA cycle becomes widely activated, leading to a more efficient ATP generation method (214). Consequently, low oxygen consumption is observed in undifferentiated stem cells in the inner cell mass, while the mtDNA copy number in trophoblast cells and mitochondrial aerobic metabolism increases (213). Interestingly, Fragouli et al.’s have shown that the mtDNA level in blastocysts significantly increases with female age, with high mtDNA levels present in 30% of non-implanting euploid embryos (215). These studies suggest that the elevated mtDNA content in blastocyst embryos of women with advanced maternal age could be driven by a compensatory mechanism (as shown in **Table 5**). This abnormal increase in mtDNA content appears to compensate for the low energy generation efficiency and diminished developmental potential observed in non-implanting euploid embryos.

**Table 5:** Prediction of embryonic developmental potential by detection of embryonic mtDNA quantity.

Patients	Number	Protocol	Technique	Results	Reference
infertile women	275 patients and 716 blastocysts	PGT-A	mtDNA copy number detection	higher mtDNA copy number in aneuploid embryos than in euploid embryos, whereas no statistically significant differences in ability to implant	(216)
infertile women	490 patients and 1505 euploid blastocysts	PGT	mtDNA copy number detection	increased implantation rates for embryos with normal and elevated mtDNA levels	(217)
infertile women	174 patients and 199 blastocysts	PGT-A	mtDNA copy number detection	increased ongoing pregnancy rate for morphologically good, euploid blastocysts, with normal/low levels of mtDNA	(218)
infertile women	829 D5 and 472 D6 blastocysts from 460 patients	PGT-A	mtDNA copy number detection	higher mean mtDNA levels in D5 than their D6 counterparts	(219)
infertile women	61 patients and 287 blastocysts	PGT-A	mtDNA copy number detection	lower mtDNA content in euploid blastocysts compared to aneuploid blastocysts	(220)

#### 4.1.2 Abnormal distribution patterns of mitochondria and increased mitochondrial vesicle complex *in vitro* matured oocytes affect development and fertilization potential

Despite the tremendous advances that have been made in IVF techniques, infertility outcomes continue to correlate strongly with the age of the patient. To date, very few clinical studies have looked at the ultrastructure of oocyte, particularly mitochondria. A study of 158 ICSI cycles evaluated the changes in mitochondrial distribution in human oocyte before and after *in vitro* maturation(IVM) and the effect of IVM on mitochondrial distribution, identifying three patterns of mitochondrial distribution: peripheral, semi-peripheral, and uniformly diffuse (221). The 64.1% of GV-stage oocyte showed a peripheral distribution, compared with 45.2% of MI oocyte, but after IVM 75.5% (80/106) of oocyte showed a uniformly spread distribution, which may explain part of the reduced developmental potential of oocyte matured *in vitro* (221).

Bianchi et al. in 2015 evaluated the ultrastructure of oocyte from patients of different ages (<35 years old and ≥35 years old, n = 36) undergoing *in vitro* aging (IVA) (due to extended culture), showing that significant decrease of mitochondria-smooth endoplasmic reticulum (M-SER) aggregates, increase of mitochondrial vesicle (MV) complexes size and amount, decrease of cortical granules and microvilli, and alterations of the spindle structure characterized both reproductive aging and IVA oocyte, these changes were significantly more evident in the reproductive aging oocyte submitted to IVA (222). M-SER aggregates are considered to be precursors of MV complexes, and they play an important role in making oocyte fertile and facilitating the formation of preimplantation embryonic developmental membranes (223). Therefore, the presence of a greater number of MV complexes in reproductively senescent oocyte is considered to be an aberration. In addition, dysregulation of the M-SER/MV ratio may also lead to disturbances in calcium homeostasis known as a cause of low fertilizing capacity of oocyte (224). Another study demonstrated that MII oocyte from IVA exhibited a low-frequency, short-duration pattern of calcium oscillations within the matrix. In human oocyte (225), oocyte with dark zona pellucida have more MVs than normal oocyte, and dark zona pellucida has been associated with reduced fertilization, implantation and pregnancy rates (226). The above studies suggest that the abnormal mitochondrial distribution pattern and increased MV complexes in our *in vitro* matured oocyte may affect fertilization capacity, but the limited number of *in vivo* matured MII oocyte in humans donated for research and ethical issues have prevented extensive studies.

In summary, limited clinical studies have shown that the copy number of oocyte and CCs mtDNA during ART can positively predict embryo quality. But the abnormal increase in mtDNA content in embryos seems to be a compensatory response to low-quality embryos. In addition, mitochondria exhibit a more peripheral distribution state, and the imbalance of M-SER/MV ratio may affect the fertilization ability of oocyte. However, due to the extremely small number of MII oocyte obtained in current clinical studies, further research is needed to confirm the above findings.

### 4.2 Aging and mitochondrial disorders affect the developmental potential of oocytes and embryos: can we intervene?

#### 4.2.1 Detection of mtDNA quantity in embryos for embryo selection

In clinical application, PGT or prenatal testing can be used to evaluate the mtDNA heterogeneity of embryos to prevent the occurrence of mitochondrial genetic diseases. According to a study, the mtDNA mutation rate of embryos should be less than 18% if they can be used for transplantation (227). However, it should be noted that PGT is not always effective, because patients carrying homoplasmic mtDNA mutations cannot be screened through PGT and mtDNA in embryos may undergo mutations during development due to environmental influences.

Practically, the assessment of mtDNA quantity accompanying the preimplantation genetic testing for aneuploidy (PGT-A) of the trophectoderm biopsy has been used to predict embryo implantation potential. Studies in multiple clinics found that high mtDNA levels in blastocysts were related to aneuploidy embryos and implantation failure (216–218). The latest retrospective investigation analyzed the transfer cycle of frozen single euploid embryos, and the results showed that no correlation was observed between mtDNA content and blastocyst morphology grades or pregnancy outcomes (219, 220). Still, lower mtDNA content was associated with delayed blastocyst development (219). It should be noted that there is currently no consensus on the threshold for the mtDNA copy number of blastocysts, and the accuracy of using mtDNA copy number as a single predictive biomarker for embryo selection and developmental potential remains low (216–220).

#### 4.2.2 Adding antioxidants to IVF or IVM culture medium to prevent oxidative stress

Some clinical trials have added compounds targeting mitochondrial function to IVF culture media in an attempt to evaluate whether they can enhance mitochondrial dysfunction caused by advanced maternal age, but it is currently unclear.

According to a report, the level of melatonin in follicular fluid is related to the quantity and quality of oocyte, and can predict the outcome of IVF (228). A clinical trial has shown that supplementing with melatonin in the IVM culture system can enhance the reactive oxygen species and Ca^2+^ levels and decrease the mitochondrial membrane potential compared to *in vivo* maturation IVF oocyte (229). In addition, compared to the control group, adding melatonin to the embryo culture medium can improve the rate of high-quality embryos on the third day in patients with repetitive low-quality embryos and the blastocyst development rate in FET patients (230). At the same time, it can increase the expression of CAT gene in blastocysts, but there is no significant statistical difference in ROS level and clinical pregnancy rate between the two groups. Another human study showed that compared to the control group, melatonin supplements increased the fertilization rate, high-quality embryo rate, and high-quality blastocyst development rate of patients with previous IVF/ICSI failures, and significantly increased the implantation rate and clinical pregnancy rate of this group of patients during FET (231).

A study demonstrated that the addition of Coenzyme Q10 supplement (MitoQ) culture during IVM of human GV-stage oocyte significantly promoted nuclear maturation and had a similar positive effect in preventing chromosomal misalignment (52).

A trial was conducted on 38 thawed embryos from 6-11 cell stages provided to 19 couples (232). Two embryos from each couple were randomly divided into two groups and cultured in a medium containing or without 1 mM L-carnitine. The results showed that adding L-carnitine to the medium significantly increased the oxygen consumption rate of morula and the formation rate of blastocysts.

Overall, current research supports the discovery that antioxidants that improve mitochondrial function may enhance pre-implantation embryo development and implantation success rates, as demonstrated by human clinical and animal studies (as shown in **Table 6**). However, since these antioxidants are not the main essential factors for pregnancy, it is even more necessary to determine whether adding them to the culture medium will have any negative effects on fetal and perinatal outcomes.

**Table 6:** Comparison of antioxidants added in IVF or IVM culture medium.

Patients	Number	Protocol	Treatment	Results	Reference
infertile women	22 patients (15 IVF vs.15 IVM/IVF oocyte)	IVF, ICSI	a melatonin-supplemented IVM/IVF system	increased the cleavage rate in the IVF versus IVM group, increased reactive oxygen species and Ca^2+^ levels, decreased mitochondrial membrane potential in IVM compared with IVF oocyte.	(229)
experiment 1:repeated-poor-quality-embryo patients; experiment 2: non-repeated-poor-quality-embryo patients	experiment 1:42 patients (48 melatonin cycles vs. 133 non-melatonin cycles); experiment 2:143 supernumerary human cleavage-stage embryos(71 in melatonin group vs. 72 in control group)	IVF	10^-7^ M melatonin added to the culture medium	increased 3 high-quality embryos in melatonin cycles, the rate of available blastocysts and clinical pregnancy rate in experiment 1; increased the expression of CAT in experiment 2	(230)
patients with repeated cycles after IVF/ICSI failure	140 patients (140 melatonin culture cycles vs. previous failed cycles)	IVF, ICSI	10^-9^ M melatonin added to the culture medium	increased the fertilization rate, cleavage rate, high-quality embryo rate, blastocyst rate, high-quality blastocyst rate, biochemical pregnancy rate and clinical pregnancy rate	(231)
infertile women	89 GV oocytes (44 in MitoQ group vs. 45 in control group)	IVM	50nM MitoQ added to the culture medium	a similar positive effect in protecting against chromosomal misalignments	(52)
infertile women	38 vitrified–thawed morulae after ICSI from 19 couples (1:1matched)	ICSI	1 mM l-carnitine added to the culture medium	increased the oxygen consumption rates of morula and the morphologically-good blastocyst formation rate	(232)

#### 4.2.3 Oral antioxidant pretreatment to improve mitochondrial function before starting IVF treatment

In IVF treatment, clinical workers have been committed to studying how to improve the quality of oocyte in order to produce more high-quality embryos for transfer to the uterus. In addition to being applied to IVF culture media, some clinical trials have used antioxidants in women with poor IVF prognosis to observe whether treatment for mitochondrial damage can improve the quality of embryos obtained.

A randomized controlled trial enrolling 169 patients in POSEIDON classification group 3 (age < 35 years, poor ovarian reserve parameters), in which participants were randomly assigned to coenzyme Q10 pretreatment or no pretreatment 60 days prior to the IVF-ICSI cycle, demonstrated that women treated with coenzyme Q10 had more high-quality embryos, usable frozen embryos, and significantly fewer women cancelled embryo transfers due to poor embryo development than the control group (233). Additional clinical trials have shown that the use of coenzyme Q10 prior to and during IVF treatment in women 31 years of age and older resulted in significant reductions in levels of follicular fluid total antioxidant capacity of mature oocyte (234).

115 patients who failed to conceive due to low fertilization rate (≤50%) in the previous IVF-ET cycle were divided into two groups in the next IVF-ET cycle: 56 patients who received melatonin treatment (3 mg/day) and 59 patients who did not receive melatonin treatment. Compared with the previous IVF-ET cycle, melatonin treatment increased fertilization rate, and the concentrations of 8-hydroxy-2'- deoxyguanosine (8-OHdG) and hexanoyl-lysine adduct in follicles were significantly reduced, which suggests that melatonin treatment can protect oocyte from free radical damage, improve mitochondria, and increase fertilization rate (235). Another clinical trial showed that supplementing with melatonin did not increase the mRNA level of the MT-ATP6 gene in CCs of ovarian follicles, as well as the likelihood of clinical pregnancy and the number of retrieved mature oocyte, but significantly reduced the number of low-quality embryos (236).

Furthermore, 214 patients who underwent previous IVF-ET and were unable to conceive received IVF-ET again after an average of 82 days of treatment with L-carnitine, and results showed that the quality of embryo on Days 3 and 5 after implantation was improved (237).

In a clinical trial of DOR patients with advanced age given resveratrol supplementation three months prior to an IVF cycle, follicular fluid was tested for 13 differentially expressed microRNAs compared to women not receiving supplementation, specifically miR-125b-5p, miR-132-3p, miR-19a-3p, miR-30a-5p and miR- 660-5p, and functional predictions of these microRNAs indicate possible regulation of mitochondrial proteins thereby controlling metabolism and mitochondrial biogenesis (238). On the contrary, a retrospective study compared the pregnancy outcomes of consecutive recipients of resveratrol supplements (200 mg/day) and a control group, showing that the clinical pregnancy rate was reduced and the risk of miscarriage was increased in the resveratrol supplemented group (239).

**Table 7** summarizes the application of oral antioxidants in the IVF/ICSI cycles. In summary, it is necessary to further study downstream signaling pathways to accurately understand how these antioxidants affect embryonic development. These molecules may not act in isolation, but rather form complex interactions. Therefore, a comprehensive approach that includes different perspectives is essential for its thorough research and application. These antioxidants are expected to be beneficial supplements to IVF treatment, especially when research evidence with larger sample sizes, multi center participation, and comprehensive long-term follow-up (including birth outcomes) is obtained.

**Table 7:** Comparison of oral antioxidants in IVF/ICSI cycles.

Patients	Number	Protocol	Treatment	Results	Reference
poor ovarian reserve women with age < 35 years old	169 patients (76 treated with CoQ10 vs. 93 controls)	IVF, ICSI	oral 200 mg CoQ10 three times a day, for a period of 60 days	increased number of retrieved oocyte, fertilization rate, high-quality embryos, available cryopreserved embryos and decreased cancelled embryo transfer rate	(233)
infertile women aged 31-46 years old	30 patients (15 treated with CoQ10 vs. 15 controls)	IVF	200 mg/day oral CoQ10	decreased follicular fluid total antioxidant capacity	(234)
infertile women with a low fertilization rate (< or =50%) in the previous IVF-ET cycle	115 patients (56 treated with melatonin vs. 59 controls)	IVF, ICSI	melatonin 3 mg/day	decreased 8-OHdG and hexanoyl-lysine adduct, increased fertilization rate	(235)
infertile women	90 patients(45 treated with melatonin vs. 45 controls)	IVF	melatonin 3 mg/day	decreased the number of low-quality embryos	(236)
patients with IVF-ET failure	214 patients(treated with l-carnitine vs. previous failed controls)	IVF, ICSI	1000 mg/day l-carnitine for 82 days on average	increased quality of embryos on Days 3 and 5	(237)
poor ovarian reserve women with advanced age	12 patients (6 treated with resveratrol vs. 6 controls)	IVF	150 mg resveratrol	increased number of fertilized good quality oocyte, decreased the level of miR-125b-5p, miR-132-3p, miR-19a-3p, miR-30a-5p and miR-660-5p	(238)
infertile women	7277 cycles (204 treated with resveratrol vs. 7073 controls)	IVF	200 mg/day resveratrol supplementation	decrease clinical pregnancy rate, increased risk of miscarriage	(239)

#### 4.2.4 Improving embryonic development potential through mitochondrial transfer technology

Mitochondrial transplantation techniques, including autologous mitochondrial transplantation, ST, and PNT, have been applied on a small scale in assisted reproductive clinical practice and live births have been reported using autologous mitochondrial transplantation and ST (70, 71, 179, 195, 196) (as shown in **Table 8**). However, mitochondrial transplantation technology still needs further optimization and development. Currently, it cannot correct fertility disorders in all elderly women by improving receptor mitochondrial function. Before considering its clinical application, more evidence is needed to demonstrate its efficiency and safety.

**Table 8:** Mitochondrial transplantation techniques in ICSI cycles.

Patients	Number	Protocol	Treatment	Result	Reference
women with multiple IVF failures	10 patients	ICSI	OPCs-derived autologous mitochondrial injection	increased fertilization rate	(70)
patients with a poor prognosis for success with standard IVF	94 patients (106 ICSI-only cycles vs. 171 AUGMENT cycles)	ICSI	OPCs-derived autologous mitochondrial injection	increased oocyte, embryo transfers, and pregnancy rate	(71)
patients with previously IVF failures	57 patients (250 ICSI-only MII vs. 253 AUGMENT MIIs)	ICSI	OPCs-derived autologous mitochondrial injection	decreased Day 5 blastocyst formation rate	(179)
women with multiple IVF failures	25 patients (28 ST cycles)	ICSI	ST	19 embryo transfers, 7 clinical pregnancies	(190)
healthy women aged 25-31 years old	32 PB1T oocytes vs. 21 control oocytes	ICSI	PB1T	decreased blastocysts rate, but similar DNA methylation and transcriptome profiles	(195)
infertile women with aged <37 years old	139 PB2T oocytes vs. 77 control oocytes	IVM, ICSI	PB2T	similar 2PN zygotes rate, cleavage embryo rate, and blastocysts rate	(196)

In summary, we summarized the close relationship between oocyte quality and mitochondria during human IVF. A certain number of mitochondria allows for normal early embryonic development and avoids untimely activation of mitochondrial biogenesis, and thus abnormalities in mtDNA content have been linked to a reduced developmental potential of the oocyte and embryo. In addition, abnormal mitochondrial distribution patterns, and abnormal structure may affect the fertilizing ability of the oocyte. A small number of clinical trials are currently attempting to measure mtDNA levels to predict embryonic developmental potential or to explore ways to improve maternal oocyte quality for better IVF outcomes through modified IVF/IVM media, oral antioxidants, and mitochondrial transplantation techniques. All of the above explorations are of great interest, and a more comprehensive understanding of the role of mitochondria in clinical cases of infertility associated with ovarian senescence will contribute to better management of this disease in the future.

## 5 Outlook

The postponement of childbirth due to the advancement of the economy and society has emerged as a worldwide concern. Maternal and fetal health risks, such as infertility, elevated miscarriage rates, birth defects, and pregnancy complications, have become significant challenges (226, 240–242). Mitochondrial function plays an important role in maintaining the physiological state of the human body, and mitochondrial damage at any stage may lead to a decrease in oocyte quality.

In this manuscript, we summarize the insights between mammalian models and human senescent oocyte and mitochondrial damage. Quantitatively, mitochondrial biogenesis is critical during oocyte maturation and fertilization. The mtDNA of the oocyte and the surrounding granulosa cells needs to be maintained at a certain quantity, and a decline in mtDNA quantity predicts a decline in ovarian reserve. Senescence-associated mtDNA instability leads to the accumulation of mtDNA mutations in oocyte, which may carry the risk of passing on abnormal mitochondria to the offspring and thus play a key role in the deterioration of oocyte quality. Excessive OS occurs in the aging ovary, and impairment of mitochondrial function is difficult to recover from by autophagy, biogenesis. The link between telomere shortening, meiotic abnormalities, apoptosis and mitochondria has also been tentatively revealed. In addition, mitochondrial dysfunction may also affect cellular communication between oocyte and surrounding GCs. Epigenetic changes also contribute to the decline in oocyte quality during ovarian aging, and the involvement of mitochondrial metabolites in the epigenetic regulation of these processes deserves further investigation. At present, some animal experiments have explored the changes in mitochondrial function during ovarian aging, but most of them have detected changes in mitochondrial related indicators. Lack of more reliable and rigorous experiments to verify the specific roles of mitochondrial related genes. At present, there is a need for more flexible application of cutting-edge molecular and cellular biology technologies to further enhance the coherence, multi-perspective, and depth of research on mitochondrial damage and ovarian aging.

Mitochondria is known as the energy factory of cells and play an important role in the maintenance of human health and the occurrence of diseases. In fact, it is difficult to accurately explain the relationship between mitochondrial damage and disease occurrence. Mitochondrial dysfunction is present in some genetic diseases, such as Perrault syndrome (243), individuals with *POLG* mutations (89, 208), and individuals with *CLPP* mutations (95, 96), all exhibiting clinical phenotypes of ovarian dysfunction and infertility. In addition, the environment in which humans is exposed, including air pollution exposure and chemical exposure, seems to be associated with premature menopause, premature ovarian failure, and low fertility (244–246). Given the sensitivity of mitochondria to the external environment, environmental pollution is likely to lead to ovarian aging by disrupting mitochondrial function. Unhealthy lifestyles, such as excessive dieting and lack of exercise, also lead to aging by affecting mitochondrial OS, biogenesis, and ATP generation (247, 248). Therefore, the mechanism of mitochondrial damage is most likely a key player in ovarian aging rather than a single pathogenic factor. The relationship between mitochondrial damage and genetics, environment, and diet deserves further research and determination. It is important to use epidemiological methods to investigate the correlation between reproductive health and mitochondrial damage, establish predictive models, and actively develop more accurate preventive measures that include the elderly population.

In addition, given that the current research on the mechanism of mitochondrial damage in ovarian aging is not comprehensive enough, emerging biomarkers that appear to predict ovarian function and embryonic development potential, such as mtDNA, may be valuable. During ART, it was found that abnormal mitochondrial distribution patterns and increased MV complexes in mature oocyte *in vitro* may affect fertilization capacity. It is feasible to predict the developmental potential of embryos by detecting the level of mtDNA in blastocysts (215). However, it should be noted that due to differences in PGT methods, biopsy methods, and techniques, it is not possible to provide standardized decision-making methods for mtDNA for embryo management. In the future, the combination of mtDNA detection with big data, multi-omics technology, and multimodal imaging may contribute to embryo management, birth defect prevention, and other aspects.

Consequently, enhancing the quality of oocyte through the improvement of mitochondrial quality appears to be a novel approach for the management and enhancement of reproductive outcomes in the elderly population. In recent years, the efficacy of targeted treatment of mitochondrial function in ART-assisted pregnancy has been discussed (249), thereby enhancing the success rate of IVF/ICSI. In a limited number of clinical studies, scholars have evaluated methods for improving oocyte energy supply through autologous mitochondrial transplantation from multiple sources (71), and reversing the decline in oocyte quality caused by aging. However, the evidence for high-level randomized controlled trials with large sample sizes from multiple centers is still very limited. We call on clinical researchers to design more rigorous trials to assess the safety and effectiveness of targeted mitochondrial therapies.

In summary, we call for more in-depth research to better understand the mechanisms and consequences of mitochondrial damage in ovarian aging. Mitochondrial targeted therapy is expected to play an important role in delaying female reproductive aging. Incorporating these new technologies and therapies into routine treatment can provide more ideal reproductive outcomes for elderly patients.

## Author contributions

WJ: Writing – original draft. YZ: Writing – original draft, Writing – review & editing. YY: Validation, Writing – review & editing. SZ: Validation, Writing – review & editing, Funding acquisition. SX: Funding acquisition, Supervision, Writing – review & editing. FL: Funding acquisition, Supervision, Writing – review & editing.

## Glossary

assisted reproductive technology: ART
*in vitro* fertilization: IVF
intracytoplasmic sperm injection: ICSI
preimplantation genetic testing: PGT
preimplantation genetic testing for aneuploidy: PGT-A
embryo transfer: ET
*in vitro* maturation: IVM
*in vitro* aging: IVA
diminished ovarian reserve: DOR
premature ovarian insufficiency: POI
premature ovarian failure: POF
follicle-stimulating hormone: FSH
anti-Mullerian hormone: AMH
germinal vesicle: GV
cumulus oocyte complex: COC
granulosa cells: GCs
cumulus cells: CCs
mitochondrial DNA: mtDNA
tricarboxylic acid: TCA
oxidative stress: OS
oxidative phosphorylation: OXPHOS
nicotinamide adenine dinucleotide: NADH
flavin adenine dinucleotide: FADH2
adenosine triphosphate: ATP
guanosine triphosphate: GTP
reactive oxygen species: ROS
reactive nitrogen species: RNS
electron transport chain: ETC
mitochondrial unfolded protein response: UPRmt
oogonial precursor cells: OPCs
cytoplasmic metastasis: CT
nuclear transfer: NT
spindle transfer: ST
pronuclear transfer: PNT
polar body transfer: PBT
mitochondrial membrane potential: MMP
mitochondria-vesicle: MV
mitochondria-smooth endoplasmic reticulum: M-SER
